# Water Fluoridation: Institutional Arrangements and Regulatory Mechanisms in Three Selected Countries

**DOI:** 10.1111/jphd.70048

**Published:** 2026-02-20

**Authors:** Paulo Frazão, Anamaria Brasilino Snellaert Tavares, Alexander J. Morris, Howard Pollick

**Affiliations:** ^1^ Department of Politics, Management and Health Public Health School, University of Sao Paulo São Paulo Brazil; ^2^ Postgraduate Program in Public Health Public Health School, University of Sao Paulo São Paulo Brazil; ^3^ School of Dentistry, University of Birmingham Birmingham UK; ^4^ Division of Oral Epidemiology and Dental Public Health, Department of Preventive and Restorative Dental Sciences School of Dentistry, University of California San Francisco San Francisco California USA

**Keywords:** fluoridation, government regulation, health policy, public health surveillance

## Abstract

**Objective:**

To describe and compare regulatory processes for quality control of community water fluoridation (CWF) in three selected countries.

**Methods:**

Documentary analysis of printed and online resources; correspondence with key informants from Brazil, the United States of America (USA), and England; countries with tradition in water fluoridation implementation for dental caries prevention.

**Results:**

The target and maximum allowable concentration of fluoride in drinking water was 0.7/4.0 mg/L in the USA; 0.7/1.5 mg/L in Brazil, and 1.0/1.5 mg/L in England. Concentration monitoring by the water companies varied; collection of samples at several points of the distribution network was required in England and the USA, while Brazil demanded only one point at the end of the water treatment. England and the USA relied on data generated by companies responsible for water treatment, while Brazil adopted an independent surveillance system that used data collected in the distribution network by the local health authority. A common point between the USA and Brazil was the construction of a nationwide information system under principles of health surveillance that could be disseminated for stakeholders. In England, annual quality reports for each water supply zone were accessible but not routinely collated and published.

**Conclusion:**

Institutional arrangements, regulatory mechanisms, and control, surveillance, and information disclosure procedures varied substantially among countries. There is potential for exchanging procedural and outcome information in order to improve the performance of fluoridation services.

## 1 Introduction

Untreated dental caries in permanent teeth is the most prevalent condition among all diseases in the world [1]. Fluoridation of drinking water to an optimal level (usually 0.5 to 1.0 mg/L) stands out as a major public health strategy for dental caries prevention. More than 400 million people distributed in more than 25 countries around the world were benefiting at the beginning of the 21st century [2].

As concentrations below 0.5 mg F^−^/L do not protect against caries and values above 1.5 mg F^−^/L increase the risk of dental fluorosis [3], the implementation of regulatory mechanisms is essential to ensure the achievement of oral health goals. To date, however, no country‐level comparative analysis has been published on the topic. Since the implementation of the first fluoride‐adjusted water treatment system in Grand Rapids, US, in 1945, many cities are now served by water utilities that have adjusted the fluoride concentration for dental caries prevention levels [2]. These water services work under distinctive regulatory arrangements translating risk‐based management policies and standard setting into both operational control and surveillance of drinking water quality [3]. Investigating the characteristics of the institutional arrangements and regulatory mechanisms adopted in different contexts can help to identify limitations and opportunities for improving practices and management of community water fluoridation (CWF).

The objective was to describe and compare the institutional arrangements and regulatory mechanisms to ensure water quality in order to guarantee the safety and effectiveness of the adjustment of fluoride concentration in three selected countries.

## 2 Methods

A descriptive study was carried out through a survey of official sources of normative documents in Brazil, the United States of America (USA), and England. These countries were selected because CWF has been used as a population strategy for more than five decades [4, 5, 6] and undesirable variation in water fluoride concentration had been documented [7, 8, 9]. Data sources were the websites of government organizations responsible for issuing and providing documents directly related to the study's focus. For this purpose, a targeted search was carried out for relevant national government agencies or authorized entities in order to obtain specific documents and details of regulations affecting drinking water supply, including quality control.

Criteria of authenticity, credibility, representativeness, and meaning were used to select documents [10]. Authenticity refers to whether the materials are original, reliable, and unaltered, and of recognized provenance. Credibility was verified by examining the authorship identity, the context in which the document was produced, and whether it contains credible data. In general, data obtained from official government documents constitute credible sources that can reveal the different institutional arrangements adopted in different countries. They are important sources of information for defining public policies, the process for decision‐making, and improving the quality of services. However, such documents may lose value over time, and it may not always be clear that they are no longer in practical use. Therefore, it is important to verify their continued relevance compared to other documents by cross referencing the information across as many sources as are accessible [10].

The content of each document was indexed in the three categories: (a) institutional arrangement defined by state form and system of government, division of the authorities' role, and regulatory model; (b) normative devices related to provision of treated water strategies and procedures for the operational control of fluoridation; and (c) strategies and procedures for water quality surveillance in relation to fluoride, data recording and dissemination of information to the public. To ensure accuracy, each country's documents were reviewed by at least two experts through a process of dialogue and active listening based on objective criteria defined by mutual agreement. Three tables show the main characteristics of each country and specific notes related to the data sources.

The study was not submitted to the Research Ethics Committee since it used official documents in the public domain.

## 3 Results

Main aspects related to institutional arrangement are summarized in Table 1. The USA [11] and Brazil [13] are constitutional republics with a three‐level federal structure, in which public drinking water services are managed by regional and local authorities through contracts between state and municipal or private entities. Economic regulation (e.g., taxation rules and mechanisms to promote company efficiency) was carried out by regulatory agencies of regional scope. On the other hand, England was part of a unitary state [12] in which services were privatized in 1989 under the economic regulation of an agency established for this purpose—the Office of Water Services (OFWAT). In common, the selected countries shared division of executive, legislative and judicial powers and existence of a central authority that, through a specific agency—Environmental Protection Agency (EPA) in the USA [14] and Drinking Water Inspectorate (DWI) in England [21], or linked directly to federal executive power—Ministry of Health (MoH) in Brazil [22], defined regulatory mechanisms to ensure control of water quality in its territory. In the USA and Brazil, state levels had complementary competences. In Brazil, local levels could also add complementary details to normative devices defined in previous levels. In Brazil, CWF was approved by federal law and maintained by the National Congress since 1974 while the decision to adjust fluoride concentration was primarily in the hands of local communities in the USA and England. In 2023, 12 states in the USA had “mandatory CWF levels” [15]. Enactment of the Health and Care Act 2022 transferred powers and responsibilities in relation to fluoridation programs to the English central authority [20].

**TABLE 1:** Main features of the institutional arrangements to ensure water supply services in the United States of America, England, and Brazil.

Categories	United States of America	England	Brazil
State form and system of government	Federated State maintained by a constitutional federal republic with separation among the executive, legislative and judiciary branches and three levels of government: federal, state, and local [11].	Member country of the United Kingdom: a unitary state maintained by a parliamentary constitutional monarchy. It is governed by the British Parliament with the separation of executive, legislative and judicial powers. The country is divided into regional authorities according to the sector of institutional action and type of public service and 386 local authorities distributed in counties whose districts may or may not have specific authority [12].	Federative state maintained by a presidential constitutional republic, with separation among executive, legislative and judicial powers and three levels of government: federal, state, and municipal [13].
Role of the Central Authority in relation to water supply	Defines norms on the quality and safety of drinking water for the population [14]. Tracks data on water quality [15]. Authorizes fluoride additives [16]. It produces and disseminates information and knowledge [17]. The EPA regulates over 160,000 water systems, and the publicly owned systems represent over 90% of all water production [18].	Water and sewage services are provided by 32 companies that were privatized in 1989 in England [19]. Since 2022 the national minister for health has had the responsibility to propose and consult on new fluoridation programs and variations to or termination of existing programs [20]. Parliament sets standards for the quality and safety of public drinking water supplies in England and Wales and a national agency (DWI) plays a central role in monitoring water quality and monitoring fluoride concentrations (natural or adjusted), and sets standards for fluoride additives used in water fluoridation. It produces and disseminates information and knowledge [21]. The UK Health Security Agency and Office for Health Improvement and Disparities (OHID) monitor the effects of water fluoridation on the health of people living in the areas covered and produce reports every 4 years on behalf of the responsible minister [6].	Defines norms on water security for sustainable development in the country and on the operational control of water quality. Defines norms on the monitoring of water quality. It produces and disseminates information and knowledge [22]. Defines the regulatory framework for the concession of basic sanitation services by municipalities [23].
States/Provinces/Regional Authorities	The economic regulatory framework extends across state, regional, and local levels through regulations by government agencies. Most operators of water supply systems are at the municipal or community level [18]. There is no federal “requirement” to fluoridate water systems: states and local communities decide whether to adjust the fluoride concentration. This includes civil entities, councils of representatives, courts, local governments, popular referendums and water authorities [18].	Takes action under national regulations that ensure the quality of privately supplied drinking water (e.g., private wells and boreholes), analogous to the actions of DWI for public water supplies [6].	Most of the water produced comes from mixed economy companies at the state level and economic regulation depends on regional agencies. Since 2020, the National Water Agency has started to define economic regulation guidelines that give greater uniformity and transparency to the rules for providing the service [23]. State government health departments are responsible for promoting and monitoring water quality surveillance, in conjunction with the Municipalities; develop actions inherent to public health laboratories; implement water quality surveillance guidelines; set priorities, goals, targets and indicators for water quality surveillance; forward to those responsible for the water supply information regarding investigations of outbreaks related to water quality. They have relevant functions to defend the interests of users [22].
Local Authorities	They apply the norms that vary according to the State. In some locations, resolutions and referendums approved by the community are adopted. They have a fundamental role in relation to CWF. They contribute to the health debate about water fluoridation in a locality and its potential to reduce health inequalities and possible alleged harms of water fluoridation [24].	Following legislation in 2022 they continue to have a key role in relation to the consideration of fluoridation through their duty to produce a strategic needs assessment for an area. They contribute to the health debate on water fluoridation in a locality and on its potential to reduce health inequalities and on possible alleged harms of water fluoridation, based on clinical plausibility and knowledge of the scientific literature [6, 20].	They carry out the surveillance guidelines for the quality of water for human consumption defined at the national and state levels. They register and authorize the supply of treated water. They inspect the quality control of produced and distributed water and the operational practices adopted. They carry out surveillance of water quality and fluoridation in their area of competence, considering regional and local peculiarities. They maintain articulation with regulatory entities when failures are detected regarding the quality of water supply services. They provide information to the population about water quality and associated health risks (outbreaks and health problems related to water quality) [22].

Regulations related to the provision of treated water, strategies, and procedures for operational control of fluoridation are summarized in Table 2. This legislation has specified, among other aspects, the roles of institutions and has defined standards in relation to equipment, fluoride sources, and measurement methods. The maximum value allowed for fluoride was 4.0 mg/L in the USA [28], while in the other countries it was 1.5 mg/L. For concentration adjustment, 0.7 mg F^−^/L was recommended in the USA, with a proposed control range of 0.6–1.0 mg F^−^/L [28], while in England the target level was 1.0 mg F^−^/L (tolerance 0.8–1.2 mg F^−^/L) [33] and values between 0.7 and 1.2 mg F^−^/L were legally required in Brazil [36].

**TABLE 2:** Main normative devices related to the provision of treated water, strategies, and procedures for the operational control of fluoridation in the United States of America, England, and Brazil.

	United States of America	England	Brazil
Water supply systems	Regulated by the Safe Drinking Water Act (1974) [14]. The 1986 and 1996 amendments to the Act and the Memorandum of Understanding (1979‐MOU 225‐78‐4006) with the FDA give the Environmental Protection Agency exclusive regulatory authority to define water safety and potability criteria [25].	Regulated by the Water Industry Act (1991) as modified by the Water Act (2003) and subsequent 2012 and 2022 Health Acts which define the requirements for the supply of water [20, 26]. The Health and Social Care Act (2012) reorganized the structure of the National Health Service in England and introduced changes to the responsibilities of local authorities and the Secretary of State for Health and Social Care in relation to operation of existing water fluoridation programs [27].	They are regulated by Law 14,026/2020, which defines the ownership of municipalities in the management of water supply services and allows the entry of the private sector in the provision of services [23].
Water fluoridation	The provision of CWF is defined by each state and locality. Once approved, the recommended level of fluoride for caries prevention purposes is 0.7 mg F^−^/L [28]. The substances, equipment and application methods used to adjust the fluoride concentration in water, including storage precautions, product contamination and worker exposure, are regulated by standards and recommendations set by the CDC and American Water Works Association (AWWA) [29, 30].	The provision of CWF is defined in accordance with the Water Industry Act (1991) [31]. The Health and Social Care Act (2012) which defined the responsibilities of the Secretary of State for Health in relation to operation of existing programs [27]. The Water Fluoridation (Proposals and Consultation) (England) Regulations (2013) established requirements on how local authorities should exercise their powers to propose, amend or terminate fluoridation programs [32]. The Health and Care Act 2022 transferred powers and responsibilities in relation to new proposals of fluoridation programs to central authority [20]. The target concentration and the performance tolerance is dictated by OHID but the tolerance values depend on arrangements between water companies and Secretary of State [33].	Law approved by the National Congress, requires the inclusion of forecasts and plans related to water fluoridation in projects intended for the construction or expansion of public water supply systems, where there is a treatment plant [34]. Decree defines appropriate fluoridation methods and processes [35]. Sets standards for fluoride additives used in water fluoridation. Concentration values can range from 0.7 to 1.2 mg F^−^/L [36].
Operational control	Operators of each water plant should be guided by state/local regulations and CDC recommendations. Monitoring of daily fluoride levels in the water distribution system is recommended [29]. Samples should be collected at points along the distribution system and points should be rotated daily. The technicians who collect the samples are responsible for ensuring that they are not contaminated and are properly labeled. At least once a month, water system personnel should share a sample. One of them must be analyzed by the operator and the other by the state laboratory or a state‐accredited laboratory [30]. All state laboratories must participate in the CDC‐sponsored Fluoride Proficiency Testing Program to ensure the accuracy of their testing program [30].	Water utilities must comply with procedures to ensure water quality in accordance with the Water Act (2003), Section 87C [37]. The required procedures are defined by a single water quality regulatory agency (Drinking Water Inspectorate). After adjusting the concentration, online monitoring should be done at the treatment plant. Operators must report the average daily volume of water produced, the average fluoride concentration value, and the percentage of time in which concentrations were within the required tolerance. In the distribution network, companies must collect samples at mixing points and reservoir outlets that receive water from a treatment plant, at a frequency per year that varies according to the volume of water in cubic meters produced per day^a^. Optionally, companies can test manually using a portable test kit for further independent verification of concentrations at different points in the water distribution network [33, 38].	Operators must annually prepare and submit for analysis by the municipal public health authority, the sampling plan for each system. They must feed information system with data on kinds of supply and on operational control. Moreover, health authorities from states and municipalities can ask for water quality control reports [22]. The control must occur at the end of the treatment. For surface sources a sample is required every 2 h, and for underground sources a weekly sample is required [39]

^a^ From 20 to 999 cubic meters, 4 samples per year were recommended; from 1000 to 49.9 thousand cubic meters, 8 samples; 50 to 89.9 thousand cubic meters, 12 samples; from 90 to 299.99 thousand cubic meters, 24 samples; from 300 to 649.9 thousand cubic meters, 36 samples; ≥ 650 thousand, 48 samples.

As for operational control, the USA and England required collecting samples from the supply network, with differences in frequency of collection. While in the first, the frequency should be daily [29], in the second it was a direct function of the size of the water supply zone [33]. In Brazil, operational control was exercised only at the end of treatment at a frequency of every 2 h for surface water sources, and weekly for underground water sources [39] (Table 2).

Procedures for water surveillance, data recording, and dissemination are summarized in Table 3. In the USA and England, water surveillance was grounded in water sample data provided by operators, while in Brazil it was supported by water samples collected from the distribution network by local health authorities. In the USA, the Centers for Disease Control and Prevention's (CDC) recommendation was that each month at least one test sample of water collected by the company responsible be analyzed by a state‐certified laboratory [30]. In England, the DWI audited water company procedures as part of its ongoing program of technical audits where the parameter fluoride was included [38]. In Brazil, the number of samples per year to be collected by local health authorities varied according to the size of the population served [40] (Table 3).

**TABLE 3:** Main strategies and procedures for water surveillance, data recording and dissemination of information in the United States of America, England, and Brazil.

	United States of America	England	Brazil
Strategies and procedures	Surveillance was carried out on the basis of audits. Data reported by operational control and the accuracy of the fluoride test equipment of the water treatment company or the laboratory were periodically verified by States and local authorities [30].	One agency (Drinking Water Inspectorate) was responsible for assessing the quality of the supply water provided, taking enforcement action (if standards are not met) and prosecuting the company which water was unfit for human consumption. The water company's arrangements for fluoridation were part of its technical audits program [38].	Based on guidelines from the Ministry of Health, local authorities were responsible for the collection and direct observation of water samples from the distribution network at a frequency per year according to the size of the population served^a^. States established laboratory references to support surveillance actions on the quality of water for human consumption. The laboratories fed Drinking Water Quality Surveillance Information System (SISAGUA acronym in Portuguese), leaving the local authorities to adopt the corrective measures with the operators [40].
Information system	*Water Fluoridation Reporting System* (WFRS) operated by the CDC in partnership with *Association of State and Territorial Dental Directors* is a data management tool. State water fluoridation program officials collect data on fluoride levels in public water systems for use by state programs and national reports. Information is provided by states on a voluntary basis and may not be reported annually [41].	Water companies must provide information to the consumer based on the postal code [42].	Drinking Water Quality Surveillance Information System is an instrument of the National Drinking Water Quality Surveillance Program (VIGIAGUA acronym in Portuguese), managed by Brazilian Ministry of Health [43].
Main data	Provides information on the status of adjusted and natural fluoridation (0.6 mg F^−^/L or more) of counties and populations served by community water systems in each state. Displays system operator‐reported fluoride level by month and number of optimally fluoridated months per year for community water systems by state [41].	Mean value of fluoride concentration. Organized by the company and/or system operator [42].	Individualized values per sample recorded by the laboratory, including collection address, date and type of supply system [44].
Update frequency and coverage	Monthly basis [41].	Quarterly and 100% of the systems [42].	Annual and coverage uninformed.
Statistical reports	Biennial [4].	Annual. DWI has offered tables with number of tests and the observed values to 1 and 99 percentiles according to each water utility [43]. A map with distribution of fluoride concentrations in the country regions [45]. The Office for Health Improvement and Disparities monitors and reports on the health effects of fluoride on people living in areas covered by water fluoridation in each 4 years [21].	Non‐periodic.
Access to user	Free	Free	Consumers do not have free access to current data of surveillance system, but an open data platform allows obtaining of crude data from earlier years [44].

^a^ For municipality with population size up to 5 thousand habitants it is recommended at least 60 samples per year; population size from 5 to < 10 thousand habitants it is recommended at least 120 samples per year; From 10 to < 15 thousand habitants, 180 samples; From 15 to < 20 thousand habitants, 216 samples; From 20 to < 30 thousand habitants, 324 samples; From 30 to < 40 thousand habitants, 432 samples; From 40 to < 50 thousand habitants, 528 samples; From 50 to < 100 thousand habitants, 636 samples and population size of 100 thousand or more habitants, 804 samples.

In relation to the dissemination of information, English water companies were responsible for making information available for the consumer which typically was available online or for those lacking IT facilities, upon request. Fluoridation data were provided to DWI by the water companies, and users could access the report on the water companies' website or access the DWI website that offered tables with the numbers of tests and values observed for the 1st and 99th percentiles [42]. A map with distributions of fluoride concentration values in each region [45] could also be obtained, and a report produced by an official agency—Public Health England—was released every 4 years describing the associations with dental and general health statistics [21] (Table 3).

Unlike the USA and Brazil, England did not have a single publicly accessible nationwide information system; annual reports for individual supply zones could be obtained from individual water companies but summative reports were not routinely collated and published. In the USA, the CDC maintained the Water Fluoridation Reporting System (WFRS) in partnership with the Association of State and Territorial Dental Directors, which provides, to registered users, information on the status of artificial and natural fluoridation (0.6 mg F^−^/L or more) of counties and populations served by each state's community water systems. The WFRS data provided the basis for the publicly available website “My Water's Fluoride” (MWF) that provides general information on fluoridated water systems. The CDC explicitly states that the MWF site does not contain real‐time information. Information is provided by states on a voluntary basis and may not be reported annually. As a result, MWF is not inclusive of all states and communities [41]. The Brazilian Ministry of Health maintained a nationwide information system on the water quality surveillance [43]. Consumers did not have free access to current data from the surveillance system, but an open data portal made it possible to obtain raw data from previous years [44]. No official document provided information on the coverage of records on fluoride concentration among Brazilian local governments. In the USA, the CDC maintained an area on its website with very detailed data and statistics on fluoridation. Annual reports on the water fluoridation coverage in the country by state and data on the average monthly concentration of fluoride in the participating water supply systems were found [41] (Table 3).

## 4 Discussion

The institutional arrangements, regulatory mechanisms, and control, surveillance, and information disclosure procedures varied substantially among countries.

Regarding the institutional arrangement, the supply, and distribution of drinking water in the USA and Brazil were carried out mostly by public companies or mixed economy (public/private) ones, while in England it was wholly by private companies. The latter had a central agency responsible for the economic regulation of the sector, establishing price/tariff parameters, levels of quality and efficiency, benefits and subsidies, among others, while the USA and Brazil had several agencies of regional scope and sectoral or multisectoral scope.

A worldwide study reported that water supplies were often regulated by direct government control or through indirect control with water companies and companies providing services of interest to the sector [46]. Among US municipalities the dynamics between municipalization and outsourcing of water services is relevant, and water distribution and treatment have one of the highest rates of stable public delivery [47]. In England, the privatization of water services in the late 1980s was surrounded by expectations for lower household costs, improved efficiency, and higher consumer satisfaction that were not realized [48]. Besides not bringing the anticipated cost‐savings, it has also been a significant barrier to expansion of fluoridation programs. In Brazil, a new regulatory framework was approved in 2007 in which local governments began to have the prerogative to manage sanitation in their jurisdiction, including changes and renewal of contracts with service providers. This new statute could increase fragmentation and commodification of the sanitation sector [5].

Decision‐making for implementing public policy also differed among countries. Similar to other public health strategies, CWF has been the subject of many debates and disputes in countries that reflect tensions between public goods and individual freedom [49]. In the USA, the decision was primarily in the hands of local communities where hearings, consultations, and referendums have been frequent. In England, ambiguity in the decision‐making process that resulted in different interpretations of the Water Fluoridation Act 1985 remained until 2003 [50]. Passage of legislation in 2022 transferred the power to propose new, or to terminate existing, water fluoridation schemes from local government to the national Secretary of State for Health and Social Care [51]. In Brazil, fluoridation has been mandatory since 1974. A proposal was presented to repeal the fluoridation law in 2003. The proposal took 13 months, passed through three commissions, and was shelved, confirming the country's option for the protection of the population oral health by the public policy [49, 52].

While more than 70% of the population served by public water supply systems benefited in the USA [4] and Brazil [5], only 10% of the English population had access to the benefit [50].

All countries had central agencies that established guidelines and regulations regarding water quality and the maximum allowable concentration of fluoride in supply systems. The concentration of 1.5 mg F^−^/L as the maximum value allowed for safe drinking water, free of contamination, whose levels are not adjusted for caries prevention purposes, was adopted in countries, according to global guidelines [3], excepting the USA that allowed up to 4.0 mg F^−^/L as the primary maximum to prevent skeletal fluorosis and 2 mg F^−^/L as a secondary maximum to prevent severe dental fluorosis. Updating the legislation on the water potability is a strategic issue for countries [53].

Regarding the recommended concentration for fluoridation systems, since 1975, Brazil has maintained a regulation with six ranges of variation depending on the average maximum daily temperature, which resulted in a range of 0.7–1.2 mg/L. In practice, the range from 0.6 to 0.8 has been adopted in most systems benefiting from the measure. Although Brazilian specialists have been demanding the updating of the regulation, the normative advice was reissued by the Ministry of Health in 2017 without complying with the technical consensus produced by the community of Brazilian professionals and researchers [54]. In England, fluoridated water supply systems have a target concentration of 1.0 mg F^−^/L (with a permitted tolerance level), while in the USA the recommended range of 0.7–1.2 mg F^−^/L changed in 2015 across the whole country to 0.7 mg F^−^/L [28].

Highly relevant finding concerns differences related to operational control procedures in each country. While the USA and England required collecting samples from the supply network, in Brazil, operational control was exercised only at the end of treatment. The requirement for controlling of fluoride concentration in situations that are closest to the conditions of use, namely in the distribution network, is important from the point of view of consumer health and for the knowledge and protection of the operator regarding eventual discrepancies with values obtained at the end of the treatment. Local situations may be made complex where multiple water sources, some fluoridated and some non‐fluoridated, whether blended or not, provide different concentrations of fluoride within a community. The criteria for determining the frequency and sample collection points associated with the volume of water produced or with the size of the population served should be replaced by characteristics of the water supply system (integrated or isolated) regarding the presence of reservoirs, points of branching and mixing, pressure differences and, mainly, the variability of observed concentration values. In case of detection of unexpected fluctuations, the inclusion of additional samples around the oscillation point is justified to verify hypotheses related to the variation. On the other hand, the greater the stability of fluoride levels in the distribution network, the smaller the number of samples required.

As for surveillance, the USA and England employed the data audit strategy, while Brazil adopted the direct observation of samples collected independently by the local health authority. In the USA, out of adjusting systems, 69% reported fluoride levels at least 1 month from 2016 to 2021 [55]. Since the 1980s, the WHO has recommended attention to the fluoride concentration by public health agencies. Global guidelines have reinforced that the main surveillance strategy to ensure water quality should be carried out by bodies that are not responsible for water treatment and distribution [3]. Surveillance initiatives carried out by local health authorities using data collected by an entity not responsible for water treatment (external control data) have been documented in Brazil since the 1990s, demonstrating their effect to improve the quality of the adjustment aiming at the maximum benefit in terms of prevention of dental caries with minimal of risk for dental fluorosis [56, 57, 58]. Compliance monitoring data should reflect the drinking water quality that consumers receive. Sampling procedures should be based on the chance that the concentration of a chemical parameter will present values outside the expected range [59]. Therefore, the frequency and collection points for the fluoride may differ from the sampling procedures determined for microbiological parameters.

Owing to low coverage of the CWF, England did not have a nationwide information system. Water companies should make information available to consumers but these data were not publicly available as shown by Moore and colleagues [5] which potentially obscured failures to deliver the program. The USA and Brazil had nationwide information systems on water quality, however, in the latter, no analysis was found on the coverage of information on fluoride concentration in water supply systems in official reports on water surveillance. Brazilian studies have shown that the completeness of data on the kinds of supply in the information system was high [60], however only 1/3 of the municipalities fed the system at least 4 months per year in relation to the fluoride concentration [9]. In the USA, information on program performance was not consistently available for all [7].

In order to allow users to monitor the level of quality of water fluoridation, it would be important to implement measures to improve data quality (scope and validity) and availability (comprehensiveness and frequency), ideally at least monthly. Brazilian specialists have claimed the dissemination of six indicators on fluoride concentration built from data obtained in each municipality. It would be a way of giving visibility to the important job of water surveillance workers and allowing longitudinal monitoring of the quality of the CWF implementation [58].

The CDC offered reports on water fluoridation coverage in the country by region every 2 years. In England, a map was made available in 4‐yearly summative reports and in Brazil no official report was published annually. This difference denotes the low priority of producing information about CWF in the Brazilian health policy agenda, even though the country in the last 65 years has managed to raise its coverage to one of the highest among the most populous countries in the world [5]. Transparency and accountability are essential principles that should be pursued both in the regulation and in the surveillance of water quality in public supply systems [3].

Studies based on documents are limited to the content of the rules and their meaning and do not allow understanding how these normative dispositions are being used in practice. In addition, documents from different countries were not prepared for research purposes, they were not produced in the same context, nor do they have the same structure, which can be a source of inconsistencies when comparing data. Despite this, the effort adopted to locate all documents relevant to the subject and rigorously distill the observed data may have minimized possible sources of error and contributed to providing a detailed description in order to give validity to the observed results. The information herein produced can help improve regulatory mechanisms and public policy management in order to reduce undesirable variations in the adjustment of fluoride concentration in water that decrease its effectiveness in achieving dental caries prevention goals. Furthermore, it may encourage studies in other countries and more specific studies within the same country where operational control and surveillance procedures are determined in a decentralized manner by subnational government authorities.

In conclusion, institutional arrangements, regulatory mechanisms and control, surveillance, and information disclosure procedures varied substantially among the three countries. The maximum allowable value for naturally occurring fluoride was 4.0 mg F^−^/L in the USA and 1.5 mg F^−^/L in other countries. England and the USA advocated collecting samples from the supply network, while Brazil only required samples at the end of the water treatment. The first two adopted different sampling criteria and used data from companies responsible for water treatment, while Brazil adopted an independent surveillance system that used data collected from the distribution network by the local health authority. The USA and Brazil have built a nationwide information system under the principles of health surveillance. Despite the differences, there is an important space for knowledge and information exchange, particularly around ways to ensure effective monitoring and maximize performance of fluoridation programs in order to achieve the public health objectives of reducing caries prevalence with minimal risk of dental fluorosis.

## Funding

This work was supported by the Brazilian Council for Scientific and Technological Development (CNPq 303447/2022‐2).

## Conflicts of Interest

The authors declare no conflicts of interest.

## Data Availability

The data that support the findings of this study were derived from official documents available in the public domain.
